# Synteny conservation between the *Prunus *genome and both the present and ancestral *Arabidopsis *genomes

**DOI:** 10.1186/1471-2164-7-81

**Published:** 2006-04-14

**Authors:** Sook Jung, Dorrie Main, Margaret Staton, Ilhyung Cho, Tatyana Zhebentyayeva, Pere Arús, Albert Abbott

**Affiliations:** 1 Department of Genetics and Biochemistry, Clemson University, Clemson, SC 29634, USA; 2 Department of Horticulture and Landscape Architecture, Washington State University, Pullman, WA 99164, USA; 3 Department of Computer Science, Saginaw Valley State University, University Center, MI 48710, USA; 4 Departament de Genètica Vegetal, Laboratori de Genètica Molecular Vegetal. CSIC-IRTA,08348 Cabrils, Spain

## Abstract

**Background:**

Due to the lack of availability of large genomic sequences for peach or other *Prunus *species, the degree of synteny conservation between the *Prunus *species and *Arabidopsis *has not been systematically assessed. Using the recently available peach EST sequences that are anchored to *Prunus *genetic maps and to peach physical map, we analyzed the extent of conserved synteny between the *Prunus *and the *Arabidopsis *genomes. The reconstructed pseudo-ancestral *Arabidopsis *genome, existed prior to the proposed recent polyploidy event, was also utilized in our analysis to further elucidate the evolutionary relationship.

**Results:**

We analyzed the synteny conservation between the *Prunus *and the *Arabidopsis *genomes by comparing 475 peach ESTs that are anchored to *Prunus *genetic maps and their *Arabidopsis *homologs detected by sequence similarity. Microsyntenic regions were detected between all five *Arabidopsis *chromosomes and seven of the eight linkage groups of the *Prunus *reference map. An additional 1097 peach ESTs that are anchored to 431 BAC contigs of the peach physical map and their *Arabidopsis *homologs were also analyzed. Microsyntenic regions were detected in 77 BAC contigs. The syntenic regions from both data sets were short and contained only a couple of conserved gene pairs. The synteny between peach and *Arabidopsis *was fragmentary; all the *Prunus *linkage groups containing syntenic regions matched to more than two different *Arabidopsis *chromosomes, and most BAC contigs with multiple conserved syntenic regions corresponded to multiple *Arabidopsis *chromosomes. Using the same peach EST datasets and their *Arabidopsis *homologs, we also detected conserved syntenic regions in the pseudo-ancestral *Arabidopsis *genome. In many cases, the gene order and content of peach regions was more conserved in the ancestral genome than in the present *Arabidopsis *region. Statistical significance of each syntenic group was calculated using simulated *Arabidopsis *genome.

**Conclusion:**

We report here the result of the first extensive analysis of the conserved microsynteny using DNA sequences across the *Prunus *genome and their *Arabidopsis *homologs. Our study also illustrates that both the ancestral and present *Arabidopsis *genomes can provide a useful resource for marker saturation and candidate gene search, as well as elucidating evolutionary relationships between species.

## Background

The eukaryote genome size is vastly diverse and is not dependent on the genetic and organismal complexity. Most of the DNA in large genomes, however, is non-coding and the gene content is relatively constant [1,2]. *Arabidopsis thaliana *(estimated haploid size of 115 Mb) contains more than 25,000 genes [3], and the Human genome (estimated haploid size of 3200 Mb) contains 20,000–25,000 genes [4]. In addition to the gene content, the conservation in the synteny (the presence of two or more genes in the same chromosome) and gene order has been observed among many plant species. One of the earliest observations of conserved macrosynteny was between potato and tomato in Solanaceae, where cDNA markers along the 12 chromosomes were largely collinear [5].

Significant conservation in the marker and gene order has been observed among grass species, despite the diverse genome size and chromosome numbers [6-8]. Similar conserved macrosynteny has also been observed in Rosaceae. Comparisons of anchor markers of the *Prunus *reference map with those of 13 maps constructed with other *Prunus *populations showed that the genomes of seven *Prunus *diploid species are essentially collinear [9]. Large collinear blocks were also detected among different genera in Rosaceae, such as *Prunus *and *Malus *[9].

On the other hand, genome sequence comparisons have revealed that plant genome evolution involved various small chromosomal rearrangements, such as insertions, deletions, inversions and translocations [10]. For example, Kilian and coworkers have shown that a barley gene in regions of high microsynteny with rice is in fact transposed to a position that is no longer syntenous with rice [11]. In addition to small chromosomal rearrangements, large segmental duplications and polyploidy is prevalent in plant genome evolution [12-14]. Genome duplication was well observed in Brassicaceae; The *Brassica *genome is extensively triplicated [15] and the *Arabidopsis *genome contains numerous large duplicated chromosomal segments [3,16]. Comparative physical mapping between *Brassica *species and *Arabidopsis *showed high conservation in the gene order but not the gene content, possibly resulting from random gene loss after extensive genome duplication in both genomes [14].

**Figure 1:**
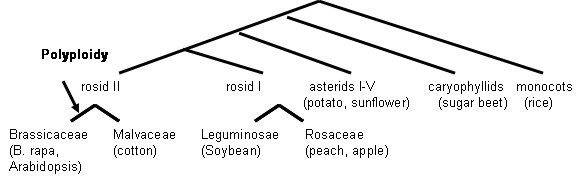
A dendrogram depicting the phylogenetic relationship of peach, *Arabidopsis *and many other crop species. The probable position of the recent polyploidization event identified from Blanc and corworkers (22) is marked by an arrow. Figure is based on Figure 1 in reference 19 and Figure 5 in reference 22.

The degree of synteny conservation has also been examined between *Arabidopsis *and less closely related species. Rosid I and rosid II comparisons (Figure 1) have been made by sequence homology between soybean marker sequences and *Arabidopsis *sequences [17]. Shared linkages were identified along with signs of extensive genome duplication and reorganization. A few microsyntenic regions were also identified by comparative physical mapping between *Arabidopsis *and soybean [18]. A gene-containing BAC sequence of tomato (asteroid I) had conserved synteny with four different segments of *Arabidopsis *chromosomes 2–5 [19].

Synteny between *Arabidopsis *and four dicotyledonous species from three major families, caryophyllids, rosids and asteroids, has also been explored by constructing genetic maps based on ESTs that are homologous to *Arabidopsis *genes [20]. Some syntenic blocks were conserved in all five maps, *Arabidopsis*, sugar beet, potato, sunflower and *Prunus*, suggesting their evolutionary significance. The syntenic blocks usually contained only several loci, however, and each linkage group of the crop genetic maps matched to multiple *Arabidopsis *genome regions. Complex syntenic relationships, suggestive of chromosome rearrangement, selective gene loss and genome duplication, were also observed [20]. Synteny between rice and *Arabidopsis *genomes, after 200 million years of divergence [21], were also observed, but the syntenic regions were scarce and separated by intervening proteins as previously suggested [20]. Also, most of the rice syntenic regions map to more than one *Arabidopsis *chromosome [21], supporting the theme of large scale genome duplication and selective gene loss in plant genome evolution.

A recent study has systematically analyzed the timing and number of segmental duplications in the *Arabidopsis *genome and suggested a recent polyploidy superimposed on older large-scale duplication [22]. The recent polyploidy appeared to have occurred during the early emergence of the Brassicaceae family and the older set of duplicated blocks between rosid I and rosid II groups. One of the interesting outcomes from this study is the reconstruction of the approximate gene order of the ancestral genome that existed prior to the recent polyploidy event. The reconstruction was done by merging genes in both sister regions duplicated at the time of polyploidy.

Rosaceae contains numerous important fruit crops such as peach, apple, cherry, pear, raspberry, blackberry and strawberry [23]. Due to the lack of availability of large genomic sequences for peach or other Rosaceae species, little information has been available to study the degree of synteny conservation between the Rosaceae species and *Arabidopsis*. A recent study has detected fragmentary macrosynteny between the *Prunus *general map and *Arabidopsis*, from comparisons of the genetic marker sequences and their *Arabidopsis *homologs [9]. When sequences of three peach genomic regions were used, only short (two or three genes) blocks that are collinear with the *Arabidopsis *genome were found [24]. With the international effort to make peach the reference species for the Rosaceae family, peach physical mapping is underway and peach ESTs are being anchored to both the genetic and physical map [25].

The objective of this study was to assess the degree of conserved synteny between *Prunus *and *Arabidopsis *using these extensive EST sequences anchored to the genetic and physical maps. We also used the reconstructed ancestral *Arabidopsis *genome to see if we coulc find additional syntenic regions. This study demonstrates that comparative genome analyses between the reconstructed *Arabidopsis *genome and other plant species can further facilitate the utilization of the genetic resources of both species and help us to understand the evolutionary relationship between these species.

## Results

### Conserved synteny between *Prunus *and *Arabidopsis*

We searched for conserved syntenic regions between the *Prunus *maps and the *Arabidopsis *genome using 475 peach ESTs anchored to the *Prunus *maps and their *Arabidopsis *homologs detected by a FASTX sequence similarity search (E value less than 10 ^-5^). The syntenic groups were selected when the distance between the two adjacent matches were less than 250 kb in the *Arabidopsis *genome and less than 10 cM in the *Prunus *maps. We detected 139 conserved syntenic regions, and 20 of them had three or more gene pairs. The number of syntenic regions between *Arabidopsis *and each of the *Prunus *maps are shown in Table 1.

**Figure 2:**
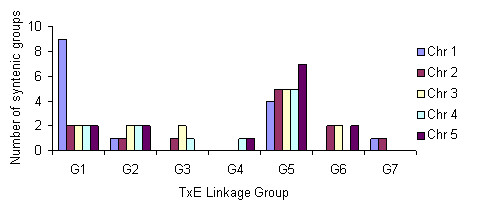
Number of syntenic groups in each TxE linkage group that match to each *Arabidopsis *chromosome.

Microsyntenic regions were detected between all five *Arabidopsis *chromosomes and seven of the eight linkage groups of the *Prunus *TxE reference map. All of the TxE linkage groups which contained syntenic regions matched to more than two different *Arabidopsis *chromosomes (Figure 2). The gene pairs in the syntenic regions showed significant sequence similarity; 78% had E values less than 10 ^-15^, and 88% had E values less than 10 ^-10^.

**Figure 3:**
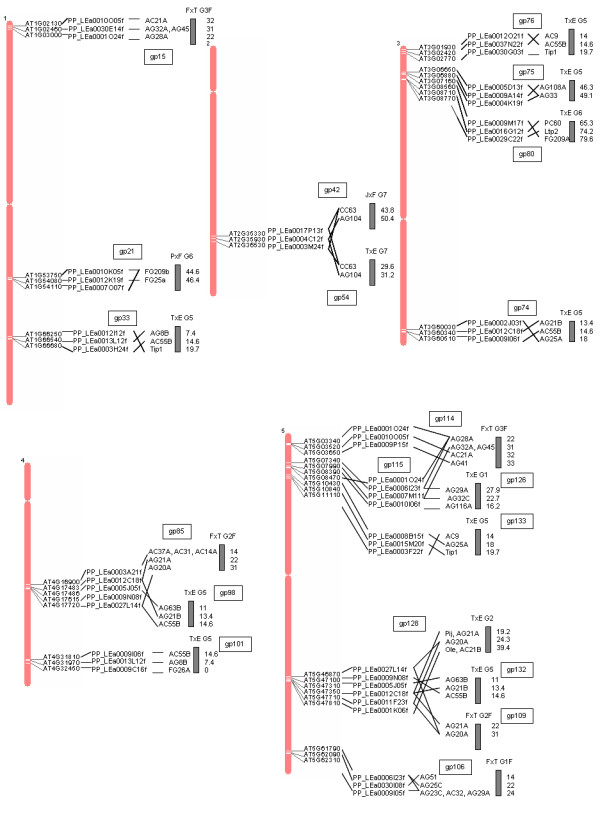
Conserved syntenic regions with three or more gene pairs between *Arabidopsis *genome and *Prunus *genome. Bolded blocks are the ones with conserved gene order.

There were 20 conserved syntenic regions with three or more gene pairs between the *Prunus *TxE map and the *Arabidopsis *genome (Figure 3). Table 2 lists these syntenic regions with the putative functions of the *Arabidopsis *genes. The largest block (group gp128) had four gene pairs, and covered 20 cM in G2 of the TxE *Prunus *map and 342 Kb in chromosome 5 of *Arabidopsis *(Figure 3). Among 20 regions with three or more gene pairs, five groups showed conserved gene order. In two groups, the collinearity could not be assessed because two different peach ESTs were anchored to the same BAC, probably by hybridizing to different gene sequences in the same BAC. In the rest of the syntenic groups, the gene order was not conserved, suggesting many chromosomal rearrangement events.

Reflecting the synteny conservation among *Prunus *maps, we detected many *Arabidopsis *regions matching to more than one *Prunus *map region. In groups gp42 and gp54, the *Arabidopsis *genes matched to the ESTs that were anchored to the same markers present in the linkage group G7 of both the TxE *Prunus *map and the JxF peach map (Table 2). In groups gp85 and gp98, the *Arabidopsis *genes within 350 kb matched to ESTs anchored to G2F of the FxT almond map and G5 of the TxE *Prunus *map (Table 2).

**Figure 4:**
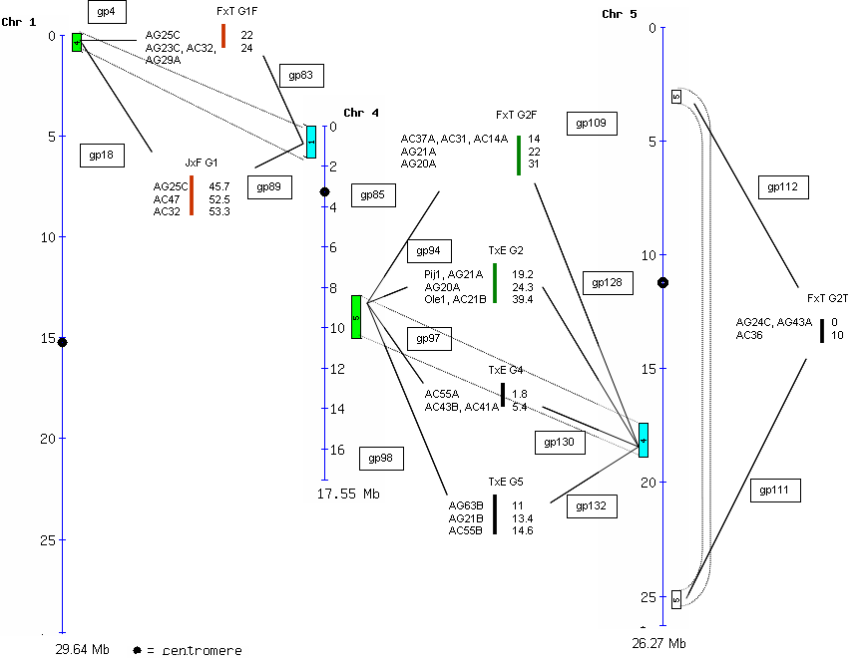
*Prunus *genomic blocks that map to two distinct *Arabidopsis *regions. Shown are the *Prunus *blocks that identified *Arabidopsis *sister regions generated by the proposed polyploidy event. The *Prunus *blocks with the same color (red or green) are homologous regions that share more than two anchored ESTs.

Most of the peach ESTs showed strong similarity to more than one *Arabidopsis *genes, and we were able to detect *Prunus *blocks that map to more than one site in the *Arabidopsis *genome. Interestingly, some of these putative duplicated *Arabidopsis *regions were located in the *Arabidopsis *paralogous blocks – duplicated blocks in a genome – reported in the previous study [21]. Figure 4 shows those *Prunus *blocks, syntenic to two different *Arabidopsis *regions, juxtaposed to the plot of the paralogous blocks of *Arabidopsis*. All three paralogons were the ones that were generated by a recent polyploidy event that occurred during the early emergence of the Brassicaceae. *Arabidopsis *blocks with conserved synteny to a region in FxT-G1F and JxF-G1 belong to the paralogons in chromosome 1 and 4, and those with conserved synteny to a region in FxT-G2T belong to the paralogons in two different arms of chromosome 5 (Figure 4). Three distinct regions in TxE – linkage groups G2, G4 and G5 – showed conserved synteny to three overlapping blocks in each paralogon on chromosome 4 and 5 (Figure 4). These TxE map regions may represent triplicated *Prunus *regions that subsequently went through selective gene loss.

### Synteny between *Prunus *and the pseudo ancestral *Arabidopsis *genome

To further analyze the evolutionary relationship between the *Arabidopsis *and *Prunus *genomes, we searched for conserved syntenic regions between *Prunus *maps and the ancestral *Arabidopsis *genome [22]. The pseudo ancestral genome contained 20187 genes, which is about 69% of the genes in the present genome, arranged in a linear array. We used the same 475 peach ESTs and their *Arabidopsis *homologs detected by FASTX sequence similarity searching (E value less than 10 ^-5^) in our search for the conserved syntenic regions. The syntenic groups were selected when the number of genes between the two adjacent matches is less than 61 in the *Arabidopsis *genome and the distance less than 10 cM of the *Prunus *maps. The estimated number of genes in 250 kb was used as the maximum distance between two matches in the *Arabidopsis *genome, since only the gene order, instead of the kb, was available as a position along the ancestral genome (see Methods).

We detected 101 conserved syntenic regions, and 12 of them had three or more gene pairs. The details, including the putative functions of the syntenic blocks with three or more gene pairs, are shown in Table 3. Fewer syntenic blocks were detected in the ancestral genome using these criteria, but much fewer blocks matched to the duplicated *Arabidopsis *genome. In the present *Arabidopsis *genome, 20 syntenic blocks, with three conserved genes, matched to 14 distinct *Prunus *regions, but, in the ancestral genome, 12 syntenic blocks matched to 10 distinct *Prunus *regions. Some groups contained the same *Arabidopsis *gene and peach EST pairs as in the syntenic groups detected from the *Prunus*-present *Arabidopsis *genome analysis. Several new *Prunus *regions were found to have conserved synteny with the ancestral *Arabidopsis *genome. The *Arabidopsis *genes in these syntenic blocks were apparently relocated in distinct regions after the putative *Arabidopsis *genome duplication event. For example, group ga54 in ancestral genome is composed of two genes in chromosome 5 and one from chromosome 3, and they were paired with ESTs that were anchored to the linkage group G1 of TxE map. Group ga28 and ga79 represent regions where three genes were closely located in the ancestral genome but they were rearranged into two different regions of the present *Arabidopsis *chromosome 5.

**Figure 5:**
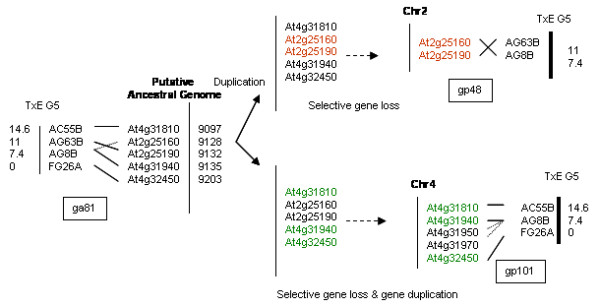
Proposed evolutionary steps involving some syntenic blocks between *Arabidopsis *and the *Prunus *genomes. Blocks in the putative ancestral *Arabidopsis *genome and *Arabidopsis *chromosome 2 and 4 that match to the same block in *Prunus *TxE map are illustrated. Red and green colors were used to help track the genes. Dashed lines were used to indicate the relationship with less stronger homology when the same EST was homologous to more than one *Arabidopsis *genes.

We also found examples where the gene content in the *Prunus *genome is more conserved in the ancestral genome than the present *Arabidopsis *genome. For example, group ga81 in ancestral genome contains four gene pairs that match to the linkage group G5 of the TxE map (Figure 5). Group gp48 and gp101 in the present genome match to the same region in TxE-G5, but contain only part of the gene pairs. Figure 5 illustrates the proposed evolutionary steps that may have occurred in these regions: large scale genome duplication and subsequent selective gene loss and gene duplication. The genomic regions in chromosome 2 and 4 were part of the previously reported duplicated regions with 68 gene pairs [22], supporting our proposed evolutionary steps.

### Synteny analysis between the peach physical transcriptome map and the *Arabidopsis *genome

We also used peach EST sequences that are anchored to the developing peach physical map to search for conserved syntenic regions between peach and *Arabidopsis*. Our data were composed of 1097 peach ESTs that are anchored to 431 BAC contigs, and their *Arabidopsis *homologs detected by FASTX sequence similarity searching (E value less than 10 ^-5^). The sequence similarity search results produced 4448 peach-*Arabidopsis *sequence pairs that consist of 904 distinct ESTs and 3747 distinct *Arabidopsis *proteins. These sequence pairs were used to detect syntenic regions between peach and *Arabidopsis*. The syntenic groups were selected when the distance between the two adjacent matches was less than 250 kb in the *Arabidopsis *genome and anchored to the same BAC contig.

Our analysis identified 287 *Arabidopsis *genes and 204 peach ESTs found in 140 syntenic blocks with at least two gene pairs. The syntenic blocks were found in all of the five *Arabidopsis *chromosomes. In peach, the syntenic blocks were found in a total of 77 BAC contigs. The synteny conservation was fragmentary; 16 out of the 18 BAC contigs with multiple syntenic regions matched to more than one *Arabidopsis *chromosome.

**Figure 6:**
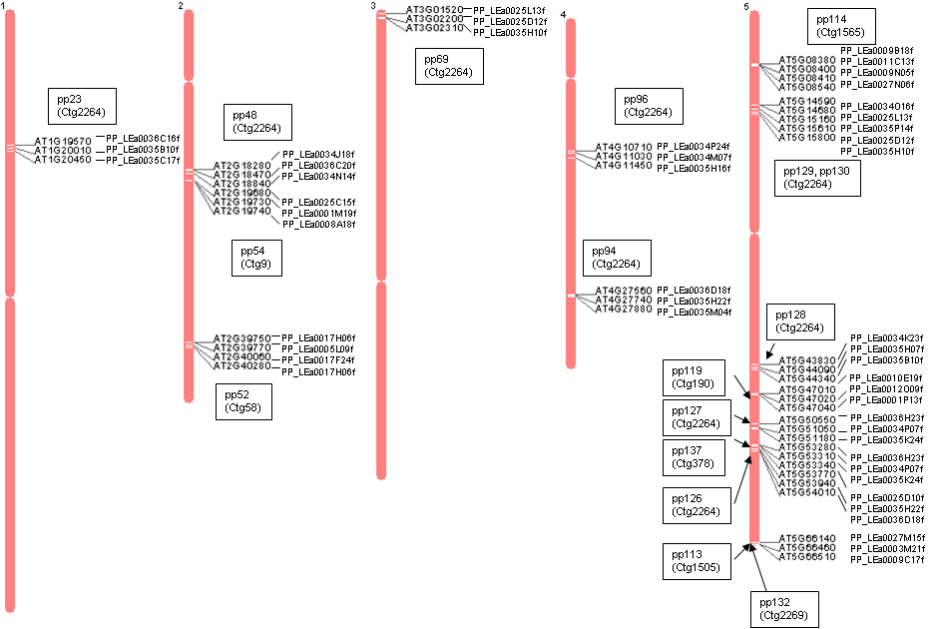
Conserved syntenic regions with three or more gene pairs between *Arabidopsis *genome and EST-anchored peach BAC contigs.

The number of gene pairs in the syntenic blocks was small: two blocks with four gene pairs, 14 blocks with three gene pairs and 124 blocks with two gene pairs. The syntenic blocks with three or more gene pairs are shown in Table 4 and Figure 6. Only two of the 16 blocks were collinear. It is possible that the content in the block is conserved but the gene order has differentially evolved in the two genomes. On the other hand, the order of the peach ESTs was estimated by the positions of the EST-hybridizing BACs in a BAC contig which may not represent the actual order of the ESTs in the genome. The average size of the syntenic blocks in *Arabidopsis *genome was 97 kb with a maximum 360 kb (group pp96: *Arabidopsis *chromosome 4 and ctg2264) and minimum 2.7 kb. Groups pp129 and pp130 were close enough to be combined into one syntenic region containing five gene pairs, and they covered 451 kb in the *Arabidopsis *genome (Figure 6).

Ctg2264 is the BAC contig that has the most anchored ESTs. It is composed of only five BACs but has 70 anchored ESTs, suggesting it represents a gene-rich region. Ctg2264 and the *Arabidopsis *genome had a number of syntenic regions including nine with three gene pairs and 22 with two gene pairs. In eight cases, the same peach EST sets in ctg2264 matched to two distinct *Arabidopsis *regions. It is notable that a relatively small contig, composed of only five overlapping BACs, had numerous microsyntenic regions found in all five *Arabidopsis *chromosomes. Ctg1502 has the second most anchored ESTs, and all the 48 anchored ESTs are limited to three BACs of the total 14 BACs composing the contig. Despite the many anchored ESTs in ctg1502, only three syntenic regions with two gene pairs were found. Only 11 of the 48 anchored ESTs had *Arabidopsis *homologs, suggesting that the rest of the ESTs may represent genes that do not exist in the *Arabidopsis *gene repertoire. However, it is also possible that we will detect more *Arabidopsis *homologs, hence more microsyntenic regions, when the entire gene sequences are available instead of short EST sequences.

In addition to the blocks in ctg2264, we found many other peach blocks corresponding to more than one syntenic region in *Arabidopsis*, reflecting the fact that the *Arabidopsis *genome contains numerous large duplicated segments [21]. In our data set, there were 21 peach segments that each corresponds to more than one distinct *Arabidopsis *segment. As expected, the *Arabidopsis *genes that matched to the same peach ESTs in these duplicated regions had similar putative function or belong to the same protein family. Some of the syntenic blocks, especially those duplicated in the *Arabidopsis *genome, were composed of genes with related function, suggesting that related genes that tend to cluster in *Arabidopsis *also do in peach. For example, all four *Arabidopsis *genes in groups pp77 and pp110 were FAD-binding domain-containing protein, similar to reticuline oxidase precursor. Similar observation has been reported in the analysis between *Arabidopsis *and rice [25]. We also observed two *Arabidopsis *segments that each corresponds to more than one distinct peach segment. Groups pp113 and pp132 involve an *Arabidopsis *region with three genes in chromosome 5 matching three peach ESTs in two different contigs (ctg1505 and ctg2269) and groups pp114 and pp123 involve an *Arabidopsis *region that matches to two different peach contigs (ctg1565 and ctg2287).

### Synteny analysis between the peach physical transcriptome map and the reconstructed *Arabidopsis *ancestral genome

The evolutionary relationship between *Arabidopsis *and peach was further analyzed by searching for conserved syntenic regions between the ancestral *Arabidopsis *genome and the peach physical transcriptome map. The syntenic groups were selected when the number of genes between the two adjacent matches was less than 61 in the *Arabidopsis *genome and anchored to the same BAC contig. This analysis identified 231 *Arabidopsis *proteins and 179 peach ESTs found in 111 conserved gene blocks. The average block size in the *Arabidopsis *genome was 27.6 genes with a maximum of 97 genes and a minimum of two genes. The estimated size of the syntenic blocks, using the average size of the *Arabidopsis *genome containing one gene per 4.1 kb (see Methods), is on average 113.2 kb with a maximum 397.7 kb and a minimum of 8.2 kb. The syntenic blocks were distributed quite evenly across the ancestral genome. In peach, the syntenic blocks were found in a total of 69 contigs. Among the 111 syntenic blocks, two blocks had four gene pairs, 12 blocks had three gene pairs and the rest had two gene pairs. The details of the 12 blocks with three or more gene pairs are shown in Table 5. Four of the 12 blocks with three or more gene pairs were collinear. Five groups contained the same *Arabidopsis *gene and peach EST pairs as those in the syntenic groups detected from the peach-present *Arabidopsis *genome analysis. Four groups involved the same regions to the ones observed in the peach-present *Arabidopsis *genome analysis, except that one or two peach ESTs were paired with *Arabidopsis *proteins from other duplicated regions. The rest of the blocks disclose peach regions that have conserved synteny with the ancestral *Arabidopsis *genome but not with the present one. In group pa3, AT5G60910 and the other two genes are closer in the ancestral genome, with only four genes in between, than in the present genome where they are 21 Mbp apart from each other. Groups pa5 and pa35 shows a similar situation in which three genes are far apart in the same chromosome of the present genome, but they are much closer in the ancestral genome.

**Figure 7:**
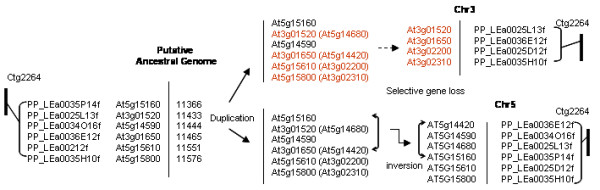
Proposed evolutionary steps involving some syntenic blocks between *Arabidopsis *and *Peach *genomes. Blocks in the putative ancestral *Arabidopsis *genome and *Arabidopsis *chromosome 3 and 5 that match to the same peach BAC contig are illustrated. Red colors were used to help track the genes. The order of the ESTs in the BAC contig was not shown because the ESTs were anchored to overlapping BACs.

Ctg2264, containing the most anchored ESTs, had one with four unordered gene pairs, four with three unordered gene pairs and 18 with two gene pairs. Upon close examination, the syntenic block with the five unordered genes observed in the present *Arabidopsis *genome (Figure 6) was also detected in the ancestral genome (Figure 7). The block was not detected from our original analysis because some of the gaps between the genes were larger than the limit set by the search parameters. The comparison revealed a syntenic block with six gene pairs in the ancestral genome and two blocks containing rearranged gene pairs in chromosome 3 and 5 of the present *Arabidopsis *genome (Figure 7). Figure 7 illustrates the proposed evolutionary steps that may have occurred in these regions: large scale genome duplication and subsequent selective gene loss in chromosome 3 and inversion in chromosome 5. Since the reconstructed ancestral *Arabidopsis *genome has been reported to contain a considerable amount of duplicated regions [22], we searched for peach EST segments that paired with more than one distinct *Arabidopsis *region. In this data set, there were eleven peach segments that each corresponds to two distinct *Arabidopsis *segments. It is notable, however, that twice as many duplicated blocks were identified by the peach EST segments in the present genome than the ancestral genome. We also observed three *Arabidopsis *segments that each corresponded to more than one distinct peach segment. Two *Arabidopsis *segments identified the same duplicated peach segments, detected from the analysis with the present *Arabidopsis *genome. Another *Arabidopsis *region identified duplicated peach regions in ctg1112 and ctg2175.

### Simulation study

To determine whether the syntenic groups we report were detected by chance, we tested the statistical significance for each group. Both the current and putative ancestral *Arabidopsis *genomes were randomized by leaving the locations the same but permuting the gene names. We analyzed 1000 simulated *Arabidopsis *genomes for the occurrence of the each conserved syntenic group and calculated the probability of the match occurring by chance. The probability of the association by chance was less than 1% for all the syntenic groups with more than three gene pairs. The numbers of syntenic groups at various significance thresholds are shown in Table 6.

## Discussion

We surveyed the degree of synteny conservation between the *Prunus *and the *Arabidopsis *genomes using extensive EST sequences anchored to several *Prunus *genetic maps and the developing peach physical map. Our study is the first to systematically examine the conserved microsynteny using DNA sequences across the *Prunus *genome and their *Arabidopsis *homologs. We could detect considerable conserved microsytenic regions even with our stringent parameters. Among the 475 genetically anchored ESTs, 142 distinct ESTs belong to the syntenic groups that were conserved with either the present or ancestral *Arabidopsis *genomes. However, the syntenic blocks were rather small in size and contained only a few gene pairs. In addition, most of the BAC contigs with more than two conserved syntenic regions matched to more than one *Arabidopsis *chromosome. Our finding is in accordance with the previous study of peach BAC sequences that the segments with a gene order congruent with *Arabidopsis *were short in any peach region studied and the corresponding segments were found in diverse locations in the *Arabidopsis *genome [24]. From the analysis with the genetically anchored ESTs, the largest block we detected had four gene pairs, and covered 20 cM in G2 of the TxE *Prunus *map and 342 Kb in chromosome 5 of *Arabidopsis*. From the analysis with the physical map-anchored ESTs, the largest block we detected contained five gene pairs and spanned 451 kb in the *Arabidopsis *genome. We may be able to find more syntenic blocks with over three gene pairs when more ESTs are hybridized to map-anchored BACs and longer BAC contigs are available. We may also find more syntenic blocks when the entire gene sequences are available. The results from the BAC contig rich in anchored ESTs, however, suggest that the syntenic regions between *Arabidopsis *and peach are typically small and contain several gene pairs at most. For example, ctg2264, with five BACs and 70 anchored ESTs, have numerous microsyntenic regions in all five *Arabidopsis *chromosomes instead of having relatively large syntenic regions.

We also detected conserved syntenic regions in the pseudo ancestral *Arabidopsis *genome that existed prior to the recent polyploidy event. We did not find markedly different results in the conserved synteny with the ancestral genome compared to the present genome, which was to be expected given that the polyploidization event that differentiated the present and the ancestral *Arabidopsis *genome occurred 24–40 million years ago, which is relatively recent compared to the peach-*Arabidopsis *divergence, 90 million years ago. We did find, however, a number of syntenic regions in the ancestral genome that do not exist in the present genome. We also found some examples where gene content and the gene order is more conserved in the ancestral genome than in the present genome. Our study illustrates that comparative genome analysis of both the ancestral and present *Arabidopsis *genomes with other plant species can provide a useful resource for marker saturation in a specific region and candidate gene searches, as well as elucidating evolutionary relationships between species.

## Conclusion

We report the results of the systematic examination of conserved microsynteny between the *Prunus *and *Arabidopsis*. Our study is the first to systematically examine the conserved microsynteny using extensive DNA sequences across the *Prunus *genome and their *Arabidopsis *homologs. More importantly, this study utilized the pseudo-ancestral *Arabidopsis *genome, as well as the present *Arabidopsis *genome, in the comparison of the *Arabidopsis *with other plant genomes. This method helped us to find more conserved microsyntenic regions between the ancestral *Arabidopsis *and *Prunus *genomes and also to delineate the putative evolutionary steps in the microsyntenic regions. We believe that this report will give a new insight in the study of evolutionary relationships among plants and provide new way to more efficient utilization of the resources of the model genome.

## Methods

### Data description

For the synteny analysis between the *Prunus *and *Arabidopsis *genomes, we used peach EST sequences anchored to the *Prunus *genetic maps [25]. Among the 475 genetically anchored peach ESTs used in this analysis, 306 ESTs were hybridized to BACs that have been hybridized to genetic markers, and the rest were hybridized to BACs belonging to a contig containing other BACs hybridized to genetic markers. The positions (cM) of the genetic markers were used as the positions for the genetically anchored ESTs.

For the synteny analysis between the peach physical transcriptome map and *Arabidopsis*, we used peach EST sequences that are anchored the developing peach physical map. The data set is composed of 1097 sequences that are anchored to 431 BAC contigs containing at least two anchored ESTs. The position of the individual BACs in the BAC contigs were used as the positions of the physical map anchored ESTs. For the ESTs that are anchored to multiple overlapping ESTs in a BAC contig, the innermost left and right positions were assigned. All the sequences and positions of the peach ESTs were obtained from the Genome Database for Rosaceae (GDR) [27,28].

The sequence data (ATH1_pep_cm_20040228) and the chromosome coordinate data (sv_gene.data) of the 29161 *Arabidopsis *translated proteins were downloaded from the *Arabidopsis *Information Resources (TAIR) database [29,30] in March 2005. The ordered list of 20187 gene names in the reconstructed ancestral *Arabidopsis *genome was downloaded from the Paralogons in *Arabidopsis thaliana *web site [22,31].

### Detection of the conserved syntenic regions

Mapped peach ESTs that are homologous to the *Arabidopsis *proteins were determined using the FASTX 3.4 algorithm [27]. Matches with E values less than 10 ^-5 ^were selected for further analysis. For the comparison between the *Arabidopsis *genome and the *Prunus *maps, the syntenic groups were selected when the distance between the two adjacent matches were less than 250 kb in the *Arabidopsis *genome and less than 10 cM for the *Prunus *maps. For the comparison between the *Arabidopsis *genome and the peach physical map, the syntenic groups were selected when the matches were located within 250 kb in the current *Arabidopsis *genome and belong to the same BAC contigs. In the analysis of the conserved synteny between the ancestral *Arabidopsis *genome and the peach physical map or the *Prunus *genetic maps, we used the estimated number of genes in 250 kb (61 genes) as the maximum distance between the two adjacent matches in the *Arabidopsis *genome. The estimation was done by dividing 250 kb by the average size per gene (4.1 kb) in *Arabidopsis*, which is derived by the division of the total length in kb by the number of genes in the *Arabidopsis *genome.

We used a program called DAGchainer [32] to detect collinear chromosomal segment conserved in the peach/*Prunus *and *Arabidopsis *genomes. DAGchainer was run with parameters set to detect any collinear blocks with two or more gene pairs and with the maximum distance between the two adjacent matches specified above. Since the DAGchainer program detects only the regions with conserved order, we developed scripts to detect both collinear and non-collinear regions from the output.

### Evaluation of the conserved syntenic regions

To determine whether the syntenic groups we report were detected by chance, we tested the statistical significance for each group. Both of the current and putative ancestral *Arabidopsis *genomes were randomized by leaving the locations the same but permuting the gene names. We analyzed 1000 simulated *Arabidopsis *genomes for the occurrence of each conserved syntenic group and calculated the probability of the match occurring by chance.

## Authors' contributions

SJ designed the protocol for synteny analysis and the statistical analysis, designed and developed scripts, performed the research, analyzed the data and wrote the paper. DM conceived of the study and participated in its design and coordination, and critically revised the manuscript. MS performed the sequence similarity search and wrote the scripts for statistical analysis. IC wrote the scripts for detecting non-linear syntenic regions and duplicate syntenic regions and parting the DAGchainer outputs. TZ provided the EST data hybridized to peach BAC contigs. PA critically revised the manuscript. AA conceived of the study and critically revised the manuscript. All authors read and approved the final manuscript.

## Figures and Tables

**Table 1:** Number of conserved syntenic regions between *Arabidopsis *and *Prunus *genetic maps.

**Map Name**	**No. anchored ESTs**	**No. Syntenic regions ** **(No. three or more gene pairs)**
^1^TxE (almond × peach)	306	68 (12)
^2^PxF (peach × peach × P. ferganensis)	188	9 (1)
^3^JxF (peach)	78	7 (1)
^4^GxN (almond × peach)	82	1 (0)
^5^FxT (almond)	171	45 (6)
^6^FxB (almond)	119	9 (0)
All Maps	475	139 (20)

^1^Dirlewanger et al. 2004 (9); ^2^Dettori et al. 2001 (33); ^3^Dirlewanger et al. 1999 (34); ^4^Jáuregui et al. 2001 (35); ^5^Joobeur et al. 2004 (36); ^6^Ballester et al. 2001 (37)

**Table 2:** Conserved syntenic regions with three or more gene pairs between the *Arabidopsis *genome and *Prunus *genetic maps.

				**Peach**	
				
**Group**	**# Pairs**	**Arabidopsis**	**Putative Function**	EST Name	Linkage Group
gp15	3	AT1G02460	glycoside hydrolase family 28 protein	PP_LEa0030E14f	FxT-G3F
		AT1G02130	Ras-related protein (ARA-5)	PP_LEa0010O05f	
		AT1G03000	AAA-type ATPase family protein	PP_LEa0001O24f	
gp21	3	AT1G53750	26S proteasome AAA-ATPase subunit (RPT1a)	PP_LEa0010K05f	PxF-G6
		AT1G54080	oligouridylate-binding protein	PP_LEa0012K19f	
		AT1G54110	cation exchanger, putative (CAX10) Ca2+	PP_LEa0007O07f	
gp33	3	AT1G66540	cytochrome P450	PP_LEa0013L12f	TxE-G5
		AT1G66250	glycosyl hydrolase family 17 protein	PP_LEa0012I12f	
		AT1G66680	S locus-linked protein	PP_LEa0003H24f	
gp42	3	AT2G35330	zinc finger (C3HC4-type RING finger) protein	PP_LEa0017P13f	JxF-G7
		AT2G35930	U-box domain-containing protein	PP_LEa0004C12f	
		AT2G36530	enolase	PP_LEa0003M24f	
gp54	3	AT2G36530	enolase	PP_LEa0003M24f	TxE-G7
		AT2G35930	U-box domain-containing protein	PP_LEa0004C12f	
		AT2G35330	zinc finger (C3HC4-type RING finger) protein-related	PP_LEa0017P13f	
gp74	3	AT3G60340	palmitoyl protein thioesterase family protein	PP_LEa0012C18f	TxE-G5
		AT3G60510	enoyl-CoA hydratase/isomerase family protein	PP_LEa0009I06f	
		AT3G60030	squamosa promoter-binding protein-like 12 (SPL12)	PP_LEa0002J03f	
gp75	3	AT3G07160	glycosyl transferase family 48 protein	PP_LEa0004K19f	TxE-G5
		AT3G06650	ATP-citrate synthase, ATP-citrate (pro-S-)-lyase	PP_LEa0005D13f	
		AT3G06880	transducin family protein	PP_LEa0009A14f	
gp76	3	AT3G02770	dimethylmenaquinone methyltransferase	PP_LEa0030G03f	TxE-G5
		AT3G01930	nodulin family protein similar to nodulin-like protein	PP_LEa0012O21f	
		AT3G02420	expressed protein	PP_LEa0037N22f	
gp80	3	AT3G08560	vacuolar ATP synthase subunit E	PP_LEa0009M17f	TxE-G6
		AT3G08710	thioredoxin family protein	PP_LEa0016G12f	
		AT3G08770	lipid transfer protein 6 (LTP6)	PP_LEa0029C22f	
gp85	3	AT4G17720	RNA recognition motif (RRM)-containing protein	PP_LEa0027L14f	FxT-G2F
		AT4G16900	disease resistance protein (TIR-NBS-LRR class)	PP_LEa0003A21f	
		AT4G17483	palmitoyl protein thioesterase family protein	PP_LEa0012C18f	
gp98	3	AT4G17483	palmitoyl protein thioesterase family protein	PP_LEa0012C18f	TxE-G5
		AT4G17486	expressed protein	PP_LEa0005J05f	
		AT4G17615	calcineurin B-like protein 1 (CBL1)	PP_LEa0009N08f	
gp101	3	AT4G32450	pentatricopeptide (PPR) repeat-containing protein	PP_LEa0009C16f	TxE-G5
		AT4G31970	cytochrome P450 family protein	PP_LEa0013L12f	
		AT4G31810	enoyl-CoA hydratase/isomerase family protein	PP_LEa0009I06f	
gp106	3	AT5G61790	calnexin 1 (CNX1)	PP_LEa0006I23f	FxT-G1F
		AT5G62310	incomplete root hair elongation (IRE)/protein kinase	PP_LEa0009I05f	
		AT5G62090	expressed protein	PP_LEa0030I08f	
gp109	3	AT5G47350	palmitoyl protein thioesterase family protein	PP_LEa0012C18f	FxT-G2F
		AT5G47710	C2 domain-containing protein contains	PP_LEa0011F23f	
		AT5G46870	RNA recognition motif (RRM)-containing protein	PP_LEa0027L14f	
gp114	3	AT5G03520	Ras-related GTP-binding protein	PP_LEa0010O05f	FxT-G3F
		AT5G03340	cell division cycle protein 48, putative/CDC48	PP_LEa0001O24f	
		AT5G03650	1,4-alpha-glucan branching enzyme	PP_LEa0009P15f	
gp115	3	AT5G07990	flavonoid 3'-monooxygenase	PP_LEa0007M11f	FxT-G3F
		AT5G07340	calnexin	PP_LEa0006I23f	
		AT5G08470	peroxisome biogenesis protein (PEX1)	PP_LEa0001O24f	
gp126	3	AT5G08390	transducin family protein	PP_LEa0010I06f	TxE-G1
		AT5G07990	flavonoid 3'-monooxygenase	PP_LEa0007M11f	
		AT5G07340	calnexin	PP_LEa0006I23f	
gp128	4	AT5G47350	palmitoyl protein thioesterase family protein	PP_LEa0012C18f	TxE-G2
		AT5G46870	RNA recognition motif (RRM)-containing protein	PP_LEa0027L14f	
		AT5G47810	phosphofructokinase family protein	PP_LEa0001K06f	
		AT5G47710	C2 domain-containing protein	PP_LEa0011F23f	
gp132	3	AT5G47100	calcineurin B-like protein 9 (CBL9)	PP_LEa0009N08f	TxE-G5
		AT5G47350	palmitoyl protein thioesterase family protein	PP_LEa0012C18f	
		AT5G47310	expressed protein	PP_LEa0005J05f	
gp133	3	AT5G10840	endomembrane protein 70, putative TM4 family	PP_LEa0015M20f	TxE-G5
		AT5G11110	sucrose-phosphate synthase	PP_LEa0003F22f	
		AT5G10430	arabinogalactan-protein (AGP4)	PP_LEa0008B15f	

**Table 3:** Conserved syntenic regions with three or more gene pairs between the pseudo-ancestral *Arabidopsis *genome and *Prunus *genetic maps.

				**Peach**	
				
**Group**	**# Pairs**	**Arabidopsis**	**Putative Function**	EST Name	BAC Contig
ga18	3	AT5G47350	palmitoyl protein thioesterase family protein	PP_LEa0012C18f	FxT-G2F
		AT4G17720	RNA recognition motif (RRM)-containing protein	PP_LEa0027L14f	
		AT5G47710	C2 domain-containing protein contains	PP_LEa0011F23f	
ga28	3	AT5G07340	calnexin, putative	PP_LEa0006I23f	FxT-G3F
		AT5G07990	flavonoid 3'-monooxygenase	PP_LEa0007M11f	
		AT5G61580	phosphofructokinase family protein	PP_LEa0001K06f	
ga29	3	AT5G14650	polygalacturonase, putative/pectinase, putative	PP_LEa0030E14f	FxT-G3F
		AT3G01610	AAA-type ATPase family protein	PP_LEa0001O24f	
		AT5G14370	expressed protein	PP_LEa0011N22f	
ga54	3	AT5G59180	DNA-directed RNA polymerase II	PP_LEa0026O17f	TxE-G1
		AT5G59840	Ras-related GTP-binding family protein epsin N-terminal homology (ENTH) domain-containing	PP_LEa0036D15f	
		AT3G46540		PP_LEa0003I01f	
ga60	4	AT2G24640	ubiquitin carboxyl-terminal hydrolase family protein	PP_LEa0006J17f	TxE-G1
		AT4G32400	mitochondrial substrate carrier family protein	PP_LEa0009H16f	
		AT2G25420	transducin family protein	PP_LEa0009H21f	
		AT2G25160	cytochrome P450	PP_LEa0013L12f	
ga66	3	AT4G17720	RNA recognition motif (RRM)-containing protein	PP_LEa0027L14f	TxE-G2
		AT5G47350	palmitoyl protein thioesterase family protein	PP_LEa0012C18f	
		AT5G47710	C2 domain-containing protein	PP_LEa0011F23f	
ga77	3	AT4G17486	expressed protein	PP_LEa0005J05f	TxE-G5
		AT5G47350	palmitoyl protein thioesterase family protein	PP_LEa0012C18f	
		AT4G17615	calcineurin B-like protein 1 (CBL1)	PP_LEa0009N08f	
ga79	3	AT5G25170	expressed protein	PP_LEa0005J05f	TxE-G5
		AT5G11110	sucrose-phosphate synthase	PP_LEa0003F22f	
		AT5G10840	endomembrane protein 70, putative TM4 family;	PP_LEa0015M20f	
ga81	4	AT4G31940	cytochrome P450	PP_LEa0013L12f	TxE-G5
		AT2G25190	expressed protein	PP_LEa0005J05f	
		AT2G25160	cytochrome P450	PP_LEa0013L12f	
		AT4G31810	enoyl-CoA hydratase/isomerase family protein	PP_LEa0009I06f	
ga83	3	AT1G66540	cytochrome P450	PP_LEa0013L12f	TxE-G5
		AT1G66250	glycosyl hydrolase family 17 protein	PP_LEa0012I12f	
		AT1G66680	S locus-linked protein	PP_LEa0003H24f	
ga94	3	AT5G58160	formin homology 2 domain-containing protein	PP_LEa0035A24f	TxE-G6
		AT5G57990	ubiquitin-specific protease 23	PP_LEa0006J17f	
		AT5G58590	Ran-binding protein 1, putative/RanBP1, putative	PP_LEa0003G19f	
ga95	3	AT5G01870	lipid transfer protein, putative	PP_LEa0029C22f	TxE-G6
		AT3G08560	vacuolar ATP synthase subunit E	PP_LEa0009M17f	
		AT3G08710	thioredoxin family protein	PP_LEa0016G12f	

**Table 4:** Conserved syntenic regions with three or more gene pairs between the Arabidopsis genome and EST-anchored peach BAC contigs.

				**Peach**	
				
**Group**	**# Pairs**	**Arabidopsis**	**Putative Function**	EST Name	BAC Contig
pp23	3	AT1G19570	dehydroascorbate reductase	PP_LEa0036C16f	ctg2264
		AT1G20010	tubulin beta-5 chain (TUB5)	PP_LEa0035B10f	
		AT1G20450	dehydrin (ERD10)	PP_LEa0035C17f	
pp48	3	AT2G18470	protein kinase family protein	PP_LEa0036C20f	ctg2264
		AT2G18840	integral membrane Yip1 family protein	PP_LEa0034N14f	
		AT2G18280	tubby-like protein 2 (TULP2)	PP_LEa0034J18f	
pp52	4	AT2G40280	Putative methyltransferase	PP_LEa0017H06f	ctg58
		AT2G39750	Putative methyltransferase	PP_LEa0017H06f	
		AT2G39770	GDP-mannose pyrophosphorylase (GMP1)	PP_LEa0005L09f	
		AT2G40060	expressed protein	PP_LEa0017F24f	
pp54	3	AT2G19740	60S ribosomal protein L31 (RPL31A)	PP_LEa0008A18f	ctg9
		AT2G19680	mitochondrial ATP synthase g subunit	PP_LEa0025C15f	
		AT2G19730	60S ribosomal protein L28 (RPL28A)	PP_LEa0001M19f	
pp69	3	AT3G02200	proteasome family protein	PP_LEa0025D12f	ctg2264
		AT3G02310	developmental protein SEPALLATA2	PP_LEa0035H10f	
		AT3G01520	universal stress protein (USP) family	PP_LEa0025L13f	
pp94	3	AT4G27880	seven in absentia (SINA) family protein	PP_LEa0035M04f	ctg2264
		AT4G27560	glycosyltransferase family protein	PP_LEa0036D18f	
		AT4G27740	Yippee putative zinc-binding protein	PP_LEa0035H22f	
pp96	3	AT4G10710	transcriptional regulator-related	PP_LEa0034P24f	ctg2264
		AT4G11450	expressed protein	PP_LEa0035H16f	
		AT4G11030	long-chain-fatty-acid – CoA ligase	PP_LEa0034M07f	
pp113	3	AT5G66460		PP_LEa0003M21f	ctg1505
		AT5G66140	20S proteasome alpha subunit D2	PP_LEa0027M15f	
		AT5G66510	bacterial transferase	PP_LEa0009C17f	
pp114	4	AT5G08400	expressed protein	PP_LEa0011C13f	ctg1565
		AT5G08380	alpha-galactosidase	PP_LEa0009B18f	
		AT5G08540	expressed protein	PP_LEa0027N06f	
		AT5G08410	ferredoxin-thioredoxin reductase	PP_LEa0009N05f	
pp119	3	AT5G47040	Lon protease homolog 1	PP_LEa0001P13f	ctg190
		AT5G47020	glycine-rich protein	PP_LEa0012O09f	
		AT5G47010	RNA helicase	PP_LEa0010E19f	
pp126	3	AT5G54010	glycosyltransferase family protein	PP_LEa0036D18f	ctg2264
		AT5G53940	Yippee putative zinc-binding protein	PP_LEa0035H22f	
		AT5G53770	nucleotidyltransferase family protein	PP_LEa0025D10f	
pp127	3	AT5G51050	mitochondrial substrate carrier family protein	PP_LEa0034P07f	ctg2264
		AT5G50550	WD-40 repeat family protein/St12p protein	PP_LEa0036H23f	
		AT5G51180	expressed protein similar to auxin down-regulated protein	PP_LEa0035K24f	
pp128	3	AT5G43830	ARG10	PP_LEa0034K23f	ctg2264
		AT5G44340	tubulin beta-4 chain (TUB4)	PP_LEa0035B10f	
		AT5G44090	calcium-binding EF hand family protein	PP_LEa0035H07f	
pp130	3	AT5G15160	bHLH family protein	PP_LEa0035P14f	ctg2264
		AT5G14680	universal stress protein (USP) family protein	PP_LEa0025L13f	
		AT5G14590	isocitrate dehydrogenase	PP_LEa0034O16f	
pp132	3	AT5G66460		PP_LEa0003M21f	ctg2269
		AT5G66510	bacterial transferase	PP_LEa0009C17f	
		AT5G66140	20S proteasome alpha subunit	PP_LEa0027M15f	
pp137	3	AT5G53280	expressed protein	PP_LEa0027O13f	ctg378
		AT5G53310	myosin heavy chain-related	PP_LEa0013H04f	
		AT5G53340	galactosyltransferase family protein	PP_LEa0003L02f	

**Table 5:** Conserved syntenic regions with three or more gene pairs between the pseudo-ancestral Arabidopsis genome and EST-anchored peach BAC contigs.

				**Peach**	
				
**Group**	**# Pairs**	**Arabidopsis**	**Putative Function**	EST Name	BAC Contig
pa3	3	AT5G07990	flavonoid 3'-monooxygenase	PP_LEa0010I09f	ctg1172
		AT5G08100	L-asparaginase/L-asparagine amidohydrolase	PP_LEa0007L05f	
		AT5G60910	agamous-like MADS box protein AGL8	PP_LEa0002N13f	
pa4	3	AT2G45560	cytochrome P450 family protein	PP_LEa0010I09f	ctg1172
		AT3G61040	cytochrome P450 family protein	PP_LEa0010I09f	
		AT2G45650	MADS-box protein (AGL6)	PP_LEa0002N13f	
pa5	3	AT1G68020	glycosyl transferase family 20 protein	PP_LEa0001F16f	ctg1172
		AT1G23870	glycosyl transferase family 20 protein	PP_LEa0001F16f	
		AT1G24260	MADS-box protein (AGL9)	PP_LEa0002N13f	
pa23	3	AT5G66510	contains bacterial transferase hexapeptide repea	PP_LEa0009C17f	ctg1505
		AT5G66140	20S proteasome alpha subunit D2	PP_LEa0027M15f	
		AT5G66460		PP_LEa0003M21f	
pa26	4	AT5G08380	alpha-galactosidase/melibiase	PP_LEa0009B18f	ctg1565
		AT5G08540	expressed protein	PP_LEa0027N06f	
		AT5G08400	expressed protein predicted proteins	PP_LEa0011C13f	
		AT5G23440	ferredoxin-thioredoxin reductase	PP_LEa0009N05f	
pa35	3	AT5G26030	ferrochelatase I	PP_LEa0004A06f	ctg1823
		AT5G11710	epsin N-terminal homology domain-containing protein	PP_LEa0003I01f	
		AT5G11770	NADH-ubiquinone oxidoreductase 20 kDa subunit	PP_LEa0001H16f	
pa37	3	AT5G47010	RNA helicase	PP_LEa0010E19f	ctg190
		AT5G47040	Lon protease homolog 1, mitochondrial (LON)	PP_LEa0001P13f	
		AT5G47020	glycine-rich protein	PP_LEa0012O09f	
pa59	3	AT4G27740	yippee family protein	PP_LEa0035H22f	ctg2264
		AT4G27880	seven in absentia (SINA) family protein	PP_LEa0035M04f	
		AT4G27560	glycosyltransferase family protein	PP_LEa0036D18f	
pa61	3	AT5G51050	mitochondrial substrate carrier family protein	PP_LEa0034P07f	ctg2264
		AT5G51180	expressed protein	PP_LEa0035K24f	
		AT5G50550	WD-40 repeat family protein/St12p protein	PP_LEa0036H23f	
pa64	3	AT4G14960	tubulin alpha-6 chain (TUA6)	PP_LEa0035B10f	ctg2264
		AT3G22170	far-red impaired responsive protein	PP_LEa0036G03f	
		AT3G22850	similar to auxin down-regulated protein ARG10	PP_LEa0034K23f	
pa71	3	AT2G18280	tubby-like protein 2 (TULP2)	PP_LEa0034J18f	ctg2264
		AT4G30260	integral membrane Yip1 family protein	PP_LEa0034N14f	
		AT2G18470	protein kinase family protein	PP_LEa0036C20f	
pa82	3	AT5G66510	contains bacterial transferase hexapeptide repea	PP_LEa0009C17f	ctg2269
		AT5G66460		PP_LEa0003M21f	
		AT5G66140	20S proteasome alpha subunit D2	PP_LEa0027M15f	
pa103	4	AT3G56080	dehydration-responsive protein-related	PP_LEa0017H06f	ctg58
		AT2G40060	expressed protein	PP_LEa0017F24f	
		AT2G39750	dehydration-responsive family protein	PP_LEa0017H06f	
		AT3G55590	GDP-mannose pyrophosphorylase	PP_LEa0005L09f	
pa108	3	AT4G29410	60S ribosomal protein L28 (RPL28C)	PP_LEa0001M19f	ctg9
		AT4G29480	mitochondrial ATP synthase g subunit family protein	PP_LEa0025C15f	
		AT2G19740	60S ribosomal protein L31 (RPL31A)	PP_LEa0008A18f	

**Table 6:** Number of syntenic groups between *Prunus*/Peach and *Arabidopsis *that are detectecd at various significance thresholds.

	Significance threshold					
	
Syntenic Group	99.90%	99%	95%	90%	80%	Total
gp	21 (17)	27 (20)	56	81	108	139 (20)
ga	11 (8)	22 (12)	39	64	86	101 (12)
pp	18 (11)	36 (16)	65	85	102	140 (16)
pa	13 (10)	25 (14)	50	70	93	111 (14)

Numbers in parenthesis stands for the syntenic groups with more than  three gene pairs.
